# The Outlet Orifice Diameter of Surgical Bioprosthetic Aortic Stented Valves Is Predominantly Much Smaller Than the Inlet Orifice Diameter

**DOI:** 10.1093/icvts/ivaf163

**Published:** 2025-07-09

**Authors:** Astrid Gerritje Maria van Boxtel, Tjark Ebels

**Affiliations:** Department of Cardio-Thoracic Surgery, University Medical Centre Groningen, 9732GZ Groningen, The Netherlands; Department of Cardio-Thoracic Surgery, University Medical Centre Groningen, 9732GZ Groningen, The Netherlands

**Keywords:** valve prosthesis, valve labelling, valve size, aortic valve prosthesis

## Abstract

**Objectives:**

Surgical prosthetic valve labelling is misleading, as labelled diameters (LDs) are currently always larger than inlet orifice diameter (IOD), while the outlet orifice diameter (OOD) is unknown. The IOD, OOD, and height of the flow channel determine its conical shape. The instructions for use (IFUs) do not list all essential metrics. This study reports a comprehensive overview of all relevant aortic stented tissue prosthetic valve metrics.

**Methods:**

We measured the OOD of these valves with an optical method. Height was measured using a calliper. The conicity angle of the flow channel was calculated. We hunted for IFU on the internet and in packaging boxes.

**Results:**

Eight valve models of 4 manufacturers were included. In all but 2 models, the OODs were smaller (89%, range 83%-95%) than their IODs, which depicts a converging shape of the flow channel. In 1 model (Avalus) OOD equals IOD, implicating a cylindrical flow channel; and 1 model (Crown) has a diverging shape. The proportion of OOD in relation to IOD seemed to be consistent among the different sizes within the same model type.

**Conclusions:**

Information on metrics for surgical aortic tissue valves is incomplete, scarce, and confusing. This article shows a comprehensive overview of valve metrics, which makes it possible to compare different aortic valve models and sizes. Flow channel shape turned out to be different amongst models. The smallest flow channel diameter is most often the OOD. Since LD should reflect the IOD, one must be aware of all relevant metrics.

## Graphical Abstract

**Figure:**
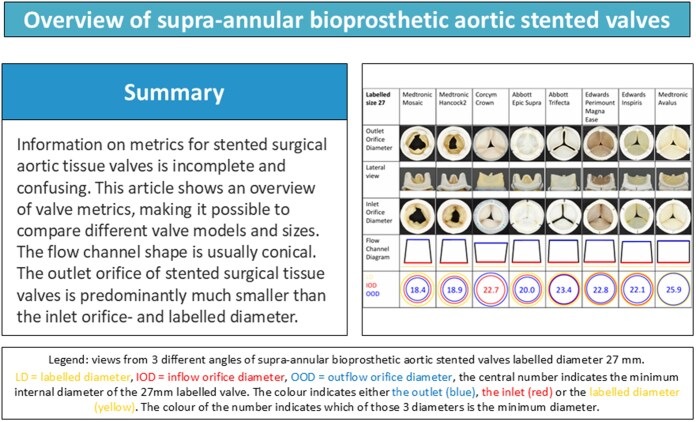

## INTRODUCTION

Surgical prosthetic valve labelling is misleading, because labelled diameters (LDs) of these prostheses are currently always larger than inlet orifice diameter (IOD). However, LD should equal patient annulus diameter (PAD), according to the applicable International Standards Organisation (ISO) standards.^1^^,^^2^ In addition, the instructions for use (IFUs) do not universally list IOD, while this is the essential metric for fitting the right size prosthesis to PAD. Furthermore, other dimensions of these prostheses are mostly not easily available in a standardized fashion.

The relationship of inlet with outlet orifice diameter (OOD) in conjunction with its height determines the shape of the flow channel. Subsequently, the diameters and conicity of the device determine the associated pressure drop. However, OOD and conicity have not been reported anywhere. Of note, OOD refers to the diameter that usually is associated with geometrical orifice area (GOA), as we investigate the entire flow channel, we choose to use OOD as being related to IOD as the limits of the flow channel.

Therefore, we measured the dimensions of these prosthetic valvar models with a novel methodology employing an advanced optical measuring device. This study reports a comprehensive overview of all relevant surgical aortic tissue prosthetic valve metrics and how these compare to each other. Subsequently, we discuss the possible implications of these findings for the haemodynamic behaviour of these prostheses.

## METHODS

We collected all available surgical supra-annular aortic stented valvar tissue prostheses currently on the market and their IFUs. We analysed all IFUs as to the availability of core metrics. We visited all manufacturer’s websites to search for relevant information on their prosthetic metrics. Meanwhile, the Abbott Trifecta valve is globally not available anymore since 2023, and the Corcym Crown valve is not available in Europe any longer.

We measured the OOD of these prosthetic valves with an optical method (Schut Geometrical Metrology) to preclude distortion of the struts by a physical measuring method, and it excludes possible friction between the valve and the measuring instrument. We observed optically the outlet of the prostheses with standardized camera settings, to determine the position of the struts and from that, we derived the OOD. We used the inside of the struts for measurement, except for the Crown and Trifecta valve. In these valves, the outside of the struts was used because their valve leaflets are mounted on the outside of the struts in contrast to the other valves.

Valve height was defined as the distance between the lower aspect of the sewing ring to the top of the strut. The height was measured using a calliper with large rectangular measuring faces. A weight of 400 g was used to standardize the pressure on the calliper in compression of the flexible sewing ring. Where surgeons are accustomed to use “height” to describe valvar prostheses, as we will do in this article, in hydrodynamics “length” is mostly used to describe the same metric.

We used our previously published data on the IODs, which were measured with the use of a conical gauge.^1^

We calculated the conicity angle of the flow channel using the formula^3^:


(Microsoft Excel code) Conicity angle=(2×ARCHTAN(((IOD−OOD)/height)/2))

We calculated the theoretical pressure drop by means of Poiseuille’s formula using the smallest diameter (SD) and compared it with LD for all available valves. We named the result the gradient multiplier, which is calculated by dividing the fourth powers of the radiuses of the SD by the LD [(SD/2)^4^/(LD/2)^4^].

Sewing ring diameters were measured with a ruler, observed and palpated; so, differences in the shapes amongst them could be described.

## RESULTS

Eight different models and all sizes of surgical supra-annular aortic valvar tissue prostheses and their IFUs were attempted to obtain (Abbott Epic Supra, Abbott Trifecta, Corcym Crown, Edwards Inspiris, Edwards Perimount Magna Ease, Medtronic Avalus, Medtronic Hancock2, and Medtronic Mosaic). IFUs were found available as printed booklets or CD-ROM in the packing boxes. All 4 manufacturers had a website but information on these sites differed substantially amongst manufacturers and changed during the observation period. In most cases, relevant information on valve metrics was very hard to find (Table 1).

**Table 1.** Instructions for Use (IFUs) Availability per Valve Model

Valve model	IFU available via website?	Where to find IFU?	**IFU available on FDA site (** https://www.accessdata.fda.gov **)**
Abbott Epic Supra	IFU available but hard to find	Home (eifu.abbott)	Yes
Abbott Trifecta	IFU available but hard to find	Home (eifu.abbott)	Yes
Corcym Crown	Only downloadable if reference number is available	Corcym Manuals	No^a^
Edwards Inspiris	Was only downloadable if reference number was available, later on it was available without this number	https://eifu.edwards.com/eifu/5d4dd5ad46e0fb0001c61ac7/DOC-0199650A.pdf	Yes
Edwards Perimount Magna Ease	Was only downloadable if reference number was available, later on it was available without this number	https://eifu.edwards.com/eifu/pages/viewers/pdf?projectKey=5d4dd5ad46e0fb0001c61ac7&itemKey=63b5f608805dc5428c946680	Yes
Medtronic Avalus	Not available, only brochure		Yes
Medtronic Hancock II	Not available, only brochure		Yes
Medtronic Mosaic	Not available, only brochure		Yes

a Also not available if the terms Livanova Crown or Sorin Crown are searched.

Hancock II with LD 19 does not exist. Our single Epic Supra valves (LD 19 and 21) and Mosaic (LD 29) were somewhat deteriorated and therefore the measurements could not be produced adequately for these sizes of these models.

Mosaic, Hancock II, and Epic Supra are porcine valves. Crown, Trifecta, Perimount, Inspiris, and Avalus are valves made of 3 bovine pericardial leaflets. Of these latter valves, in 3 models the pericardium is mounted inside the struts (Perimount, Inspiris, and Avalus) and in 2 others the pericardium is mounted on the outside of the strut (Crown and Trifecta). In most valves the 3 valve leaflets are equal in size, but in the Mosaic and Hancock II valves, one of the leaflets is larger than the other 2.

**Figure 1.**
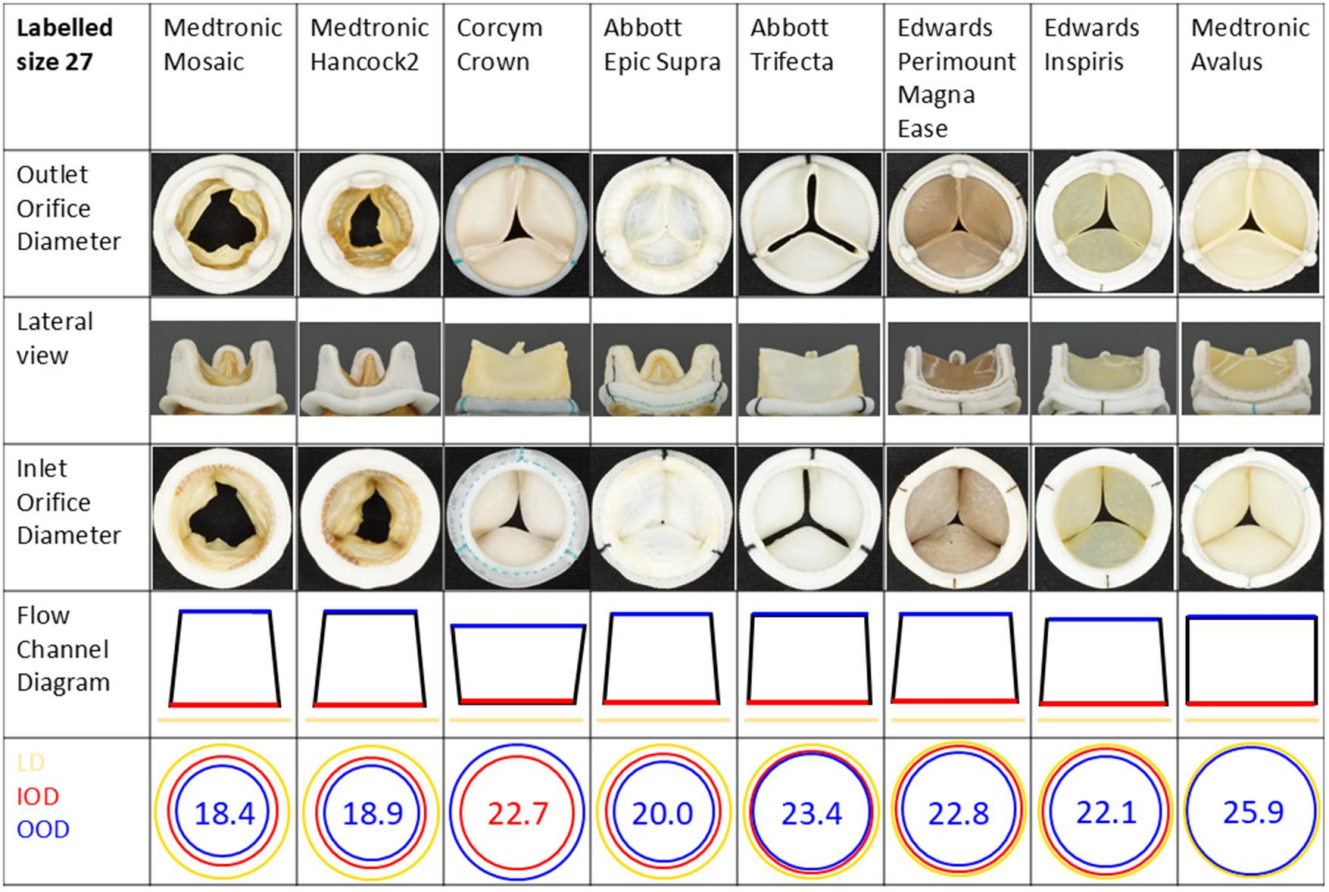
Overview of Supra-annular Bioprosthetic Aortic Stented Valves. Overview of different views of supra-annular bioprosthetic aortic stented valves labelled diameter 27 and their corresponding diameters

**Figure 2.**
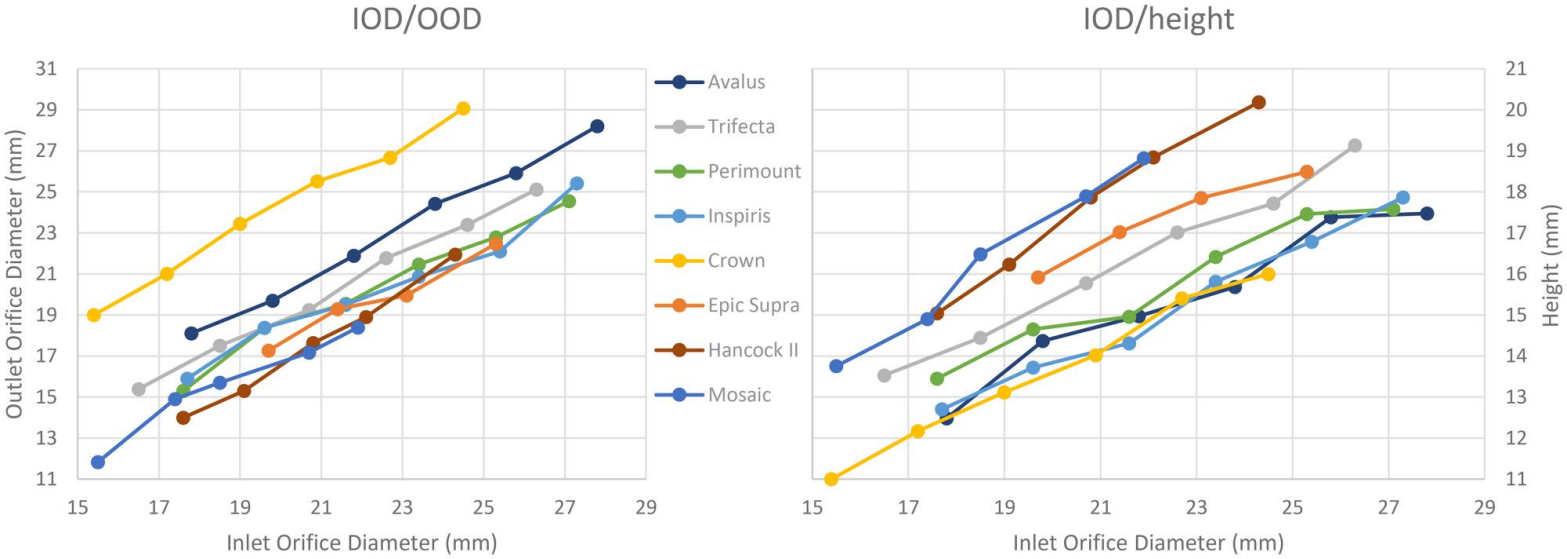
Graph Showing the Relation Between Inlet Orifice Diameter (IOD) and Outlet Orifice Diameter (OOD), and Height per Valve Model. (A) Relation between IOD and OOD per valve model. (B) Relation between IOD and height per valve model

In all but 2 models, the OODs were smaller (89%, range 83%-95%) than their corresponding IODs (Table 2), which means the flow channels of 6 of 8 models have a converging conical shape (Figure 1). Exceptions are the Medtronic Avalus and the Corcym Crown; for the Avalus valve the IOD and OOD are virtually identical, so the flow channel could be regarded to be quasicylindrical. For the Crown valve OOD was 21% larger than IOD, so the flow channel is a diverging conus. The proportion of OOD in relation to IOD seemed to be consistent among the different sizes within the same model type (Figure 2).

**Table 2.** Relevant Valve Metrics per Model

Labelled diameter (mm)	Inlet orifice diameter (mm)	Outlet orifice diameter (mm)	Height (mm)	Cone angle (°)
**Abbott Epic Supra**				
19	15.9			
21	17.7			
23	19.7	17.3	15.9	8.8
25	21.4	19.3	17.0	7.1
27	23.1	20.0	17.0	10.1
29	25.3	22.5	18.5	8.7
**Abbott Trifecta**				
19	16.5	15.4	13.5	4.7
21	18.5	17.5	14.5	3.9
23	20.7	19.2	15.8	5.3
25	22.6	21.8	17.0	2.8
27	24.6	23.4	17.7	3.9
29	26.3	25.1	19.1	3.5
**Corcym Crown**				
19	15.4	19.0	11.0	−18.6
21	17.2	21.0	12.2	−17.8
23	19	23.4	13.1	−19.2
25	20.9	25.5	14.0	−18.7
27	22.7	26.7	15.4	−14.7
29	24.5	29.1	16.0	−16.3
**Edwards Inpiris**				
19	17.7	15.9	12.7	8.1
21	19.6	18.4	13.7	5.1
23	21.6	19.5	14.3	8.4
25	23.4	20.9	15.8	9.2
27	25.4	22.1	16.8	11.2
29	27.3	25.4	17.9	6.0
**Edwards Perimount Magna Ease**				
19	17.6	15.3	13.5	9.8
21	19.6	18.4	14.7	4.8
23	21.6	19.5	15.0	7.9
25	23.4	21.5	16.4	6.8
27	25.3	22.8	17.5	8.2
29	27.1	24.5	17.6	8.3
**Medtronic Avalus**				
19	17.8	18.1	12.5	−1.4
21	19.8	19.7	14.4	0.4
23	21.8	21.9	15.0	−0.4
25	23.8	24.4	15.7	−2.3
27	25.8	25.9	17.4	−0.4
29	27.8	28.2	17.5	−1.3
**Medtronic Hancock II**				
19	Non existent			
21	17.6	14.0	15.0	13.7
23	19.1	15.3	16.2	13.3
25	20.8	17.6	17.9	10.1
27	22.1	18.9	18.8	9.7
29	24.3	22.0	20.2	6.7
**Medtronic Mosaic**				
19	15.5	11.8	13.8	15.2
21	17.4	14.9	14.9	9.6
23	18.5	15.7	16.5	9.7
25	20.7	17.2	17.9	11.3
27	21.9	18.4	18.8	10.7
29	23.7			

Valve heights differed from 11 mm for the Corcym Crown 19 valve to 20 mm for the Hancock II 29 valve. Ratios between heights and IODs generally decreased with increasing diameter for 6 of 8 valve models (Table 2, Figure 2). In the Hancock II and Mosaic models, this ratio was similar for each diameter.

**Figure 3.**
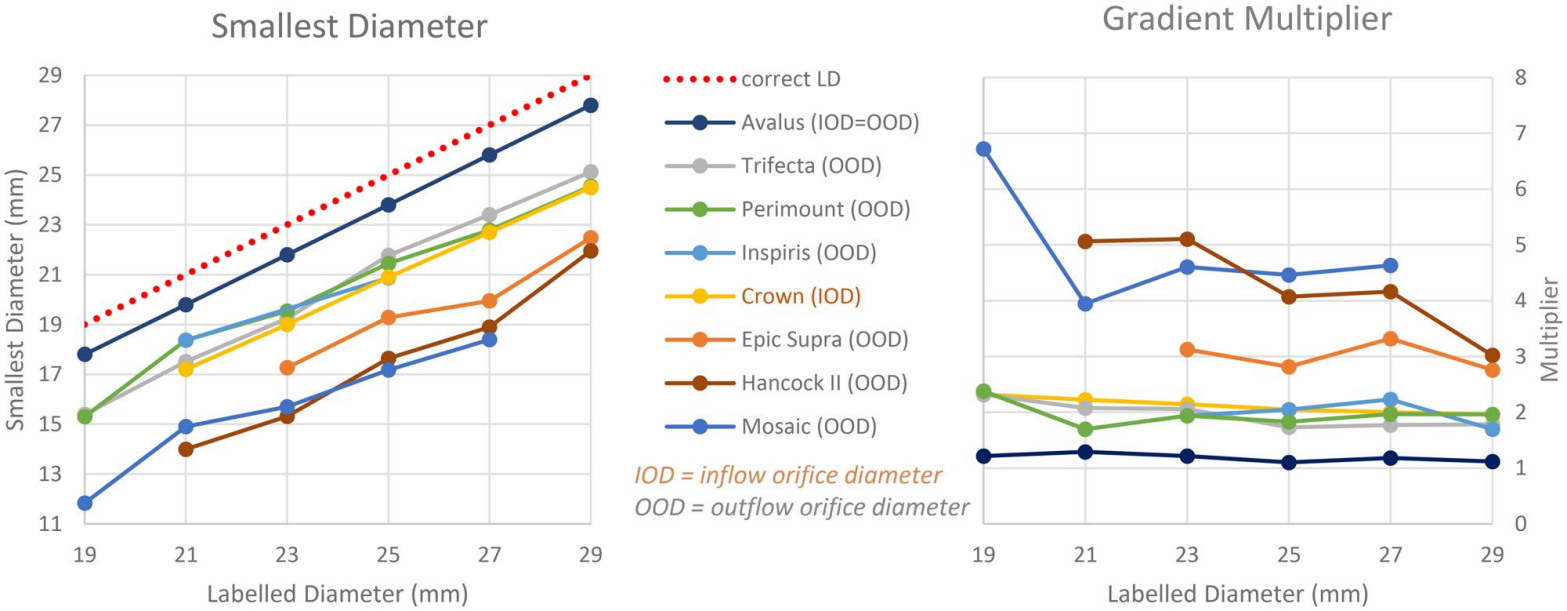
Difference Between Labelled Valve Diameters and Smallest Valve Diameters and Its Effect on Gradient Multiplier per Valve Diameter and Model. (A) Relation between labelled diameter and smallest diameter. (B) Relation between labelled diameter and number of times an initial gradient should be multiplied (gradient multiplier) to correct for adequate diameter

Porcine valves had a higher gradient multiplier (2.8-6.7) than bovine valves The Mosaic valve with LD 19 was an outlier with a multiplier of 6.7. The Avalus valve had the smallest gradient multiplier, which was 1.1 (Figure 3).

The struts of the porcine valves were bulkier and broader than those of the pericardial valves. The Avalus valve had a kind of cushion-like structure on the outside of the struts (Figure 1). The heights of the struts differed, among valve models and sizes, which leads to a variable protrusion of the prostheses into the aortic root.

Sewing rings were all circular but differ in 3D shape. Some of the valves had a “flat” sewing ring in relation to the axis of flow (Crown, Trifecta, and Avalus), where others had a curvy shape (Mosaic, Hancock II, Epic Supra, Perimount, and Inspiris), reflecting the expected 3D shape of the tri-leaflet aortic annulus. Valves shaped to fit onto a bi-leaflet valve are not available. Sewing ring width differs amongst valves; the Perimount has the smallest sewing ring (3 mm) and several other valves (Epic, Hancock II, and Crown) the largest (5 mm). In some of the valves sewing ring width differs per size (Crown), the larger the labelled size, the larger sewing ring width, but in most of the valves this width is equal for each size of the same valve model. Two valves lack indicator marking (Mosaic and Hancock II), 3 valves have indicator markings on the sewing ring adjacent to the struts (Crown, Epic Supra, and Trifecta) and 3 have indicator markings near the nadirs (Perimount, Inspiris, and Avalus). The sewing ring of the Epic Supra is mounted in such a fashion that it points upwards and it seems easier to insert the valve in an intra annular position, since the inner border of the sewing ring is mounted lower than the outer (Figure 1). The other sewing rings are mounted below the valves and can be folded upwards or downwards so the valve can be placed in either intra- or supra-annular position. Sewing rings are made of polyester (Abbott, Medtronic), polytetrafluoroethylene (Edwards), or flexible tungsten loaded silicone (Corcym). Abbott and Edwards enclose a silicone ring (Figure 1).

## DISCUSSION

The novel optical method enabled us to measure OOD without touching the flexible valve. Therefore, this measuring method did not influence the metric itself. Other physical methodologies might influence the position of the struts and thereby result in a flawed measurement. Our previous study^1^ focused on valve diameter labelling practices which are related to IOD diameters. In contrast, this study deals with the smallest internal diameter and the resulting flow channel shape. Those diameters could not be measured using the same methodology, therefor we switched from the conical gauge method to the optical method for OOD measurement. IOD measurements could not be measured using this novel method because then the closed leaflets preclude using that methodology.

Typically, the final important purpose of aortic valve replacement is preservation of the left ventricular function. For this reason, one of the most important factors is a minimal pressure gradient between the left ventricle and the aorta. That is also why pressure gradient is an important measure in cardiac ultrasound follow-up of aortic valve patients. To limit this pressure gradient to a minimum, the diameter, length, and shape of the passage from the left ventricle to the aorta through the aortic valve are of utmost importance. Interestingly, there is no publication describing the shape of the flow channel in these valves. The flow channel in most valves has a converging conical shape, except for the Avalus that is cylindrical and the Crown where that sports a diverging conical shape. As a consequence, this means that usually not the IOD but the OOD is the SD where through blood passes. The proportions between OOD and IOD differ per model and per size, but in a directly proportional manner (Figure 2). Unfortunately, the conicity showed to have the largest angle mostly in the smaller sizes, which makes those OODs even relatively smaller in comparison to the IODs.

Assuming that the IOD is equal to PAD in an adequately chosen valvar prosthesis, turbulence, if any, should not start at the level of the IOD. On the contrary turbulence, if any, should start at the OOD. Therefore, listing of the OOD amongst other relevant metrics should be stimulated, as the smaller OOD is the haemodynamically limiting factor in these valves.

The diameter has a fourth power effect on the flow according to Poiseuille’s formula. Therefore, we calculated the effect of the SD on the theoretical pressure drop. For an example, one could take 27 mm as the desired IOD as this has to fit on the congruent PAD. So assuming a 27-mm valve is necessary for a specific patient the pressure drop multiplier varies from 1 in the Avalus to 4.5 in the Mosaic prosthesis (Figure 3).

Furthermore, one must be aware that the apparent shape of the flow channel may differ from the outside aspect of the valve (Figure 1). Valve shape theoretically influences the relationship between GOA and effective orifice area (EOA).^4^^,^^5^ The diameter associated with EOA is usually distal to the OOD, and due to the phenomenon of flow contraction even smaller than the OOD.^6^

The fluid flow through a converging channel results in an increase in the outlet velocity (Bernoulli’s theorem) and consequently, a pressure drop greater than in a cylindrical channel. In addition, a narrow outlet diameter of any flow channel causes a phenomenon called “flow contraction,” which means a further narrowing in the high-velocity fluid stream downstream of the narrowing. If the IOD constitutes a decrease in the bloodstream in relation to the anatomical outflow tract upstream from the prosthetic valve, flow contraction will be the result, irrespective of OOD diameter. The advantage of supra-annular valvar prostheses is that surgeons can choose a prosthesis of equal diameter as the aortic annulus.

The height of the valve determines the length of the flow channel. This is an important metric because this length is proportional to the pressure drop, according to the Poiseuille equation. The fact that in most valves (except for Hancock II and Mosaic) the ratios between heights and IODs increase with decreasing valve sizes adds to a larger pressure drop in smaller valves. So apart from the valve being smaller in diameter, it is relatively higher, adding to the pressure drop.

In addition, a relatively lower valve is associated with a flow shape that continues to accelerate and converge after it exits through the restrictive outlet, and the EOA is then more distal and smaller than the GOA.^7^

It turned out that porcine valves had a higher gradient multiplier than bovine valves (Figure 3). This can be explained by the fact that their LDs deviate most from their actual SDs. The Avalus valve had the smallest gradient multiplier because it is the valve which is most accurately labelled and has an cylindrical shape.

Corcym Crown and St Jude Trifecta valves differ from the other valves because they have mounted their leaflets on the outside of the struts. This results in larger OODs and favourable haemodynamic parameters related to that.^8–10^ Unfortunately, these valves are known to have a limited durability.^11^ Possible explanation for this could be the fact that the leaflets are mounted on the outside of the struts, but this study also shows that the shape of the flow channel of these valves differs. This shape could conceivably be related to deterioration of the valve. Therefore, scientifically, this observation is relevant to future design considerations of these valvar prostheses.

The ISO Standard #5840 on “Surgically implanted heart valve substitutes” describes that LD should equal IOD in supra-annular prosthetic valves.^2^ In reality, all LDs are larger than IODs.^1^ On top of that, this article shows that in the majority of the valves IOD is not the SD, which is the OOD instead. Therefore, one must be aware that labelled valve diameter does not reflect the smallest flow channel diameter. This forms a risk for patient prosthesis mismatch (PPM), which is a serious danger for the patient.^12^  Table 3 shows a useful overview for surgeons showing the smallest and largest available valve diameter for PAD, which is finally the determinative diameter onto which (the IOD of) the valve has to be fitted. Figure 3 depicts the difference between labelled valve diameters and smallest valve diameters and its effect on gradient multiplier for every model. Those parameters are directly related to each other and therefore show a similar pattern. Nevertheless, they are presented in both ways because the valve diameter is a very relevant metric for the technical aspects for the surgeon, while gradient is the relevant outcome measurement for the patient.

**Table 3.** Valve Models and Sizes per Patient Annulus Diameter^a^

PAD or IOD (mm)		Medtronic Avalus	Corcym Crown	Edwards Inspiris	Edwards Perimount Magna Ease	Abbott Trifecta	Abbott Epic	Medtronic Mosaic		Medtronic Hancock
15 (14.1-16.0)	IOD		*15.4*				**15.9**	15.5		
	OOD		19.0					***11.8***		
	LD		19				19	*19*		
17 (16.1-18.0)	IOD	**17.8**	17.2	17.7	17.6	*16.5*	17.7	17.4		17.6
	OOD	18.1	21.0	15.9	15.3	15.4		14.9		***14.0***
	LD	19	21	19	19	19	21	21		*21*
19 (18.1-20.0)	IOD	19.8	19.0	19.6	19.6	*18.5*	19.7	*18.5*		19.1
	OOD	**19.7**	23.4	18.4	18.4	17.5	17.3	15.7		***15.3***
	LD	21	23	21	21	21	23	23		*23*
21 (20.1-22.0)	IOD	**21.8**	20.9	21.6	21.6	*20.7*	21.4	*20.7*	21.9	20.8
	OOD	21.9	25.5	19.5	19.5	19.2	19.3	***17.2***	18.4	17.6
	LD	23	25	23	23	23	25	*25*	27	25
23 (22.1-24.0)	IOD	**23.8**	22.7	23.4	23.4	22.6	23.1	23.7		*22.1*
	OOD	24.4	26.7	20.9	21.5	21.8	20.0			***18.9***
	LD	25	27	25	25	25	27	29		*27*
25 (24.1-26.0)	IOD	**25.8**	24.5	25.4	25.3	24.6	25.3			*24.3*
	OOD	25.9	29.1	22.1	22.8	23.4	22.5			***22.0***
	LD	27	29	27	27	27	29			*29*
27 (26.1-28.0)	IOD	**27.8**		27.3	27.1	*26.3*				
	OOD	28.2		25.4	***24.5***	25.1				
	LD	29		29	*29*	29				

Abbreviations: IOD, inlet orifice diameter; LD, labelled diameter; OOD, outlet orifice diameter; PAD, patient annulus diameter.

a Underlined numbers indicate largest diameter for this PAD; *Italic numbers* indicate smallest diameter for this PAD; bold numbers indicate largest/smallest diameter for this valve.

Our search results show that there is insufficient information available on websites, IFUs, and downloadable folders. Necessary information is very hard to find and not presented in a structural manner. The information in the box is not effective as it is usually only available after the valve has been purchased, while the information should be available prior to purchasing the valve. On one hand this is necessary to compare valves, on the other hand to be prepared for the operation. Next to this, for many of the valves (Corcym and Edwards) one needs packaged reference numbers to download the IFU. This also means that one has to buy the prosthesis before one is able to read up on its features. Furthermore, some information is incorrect and/or incomplete. What further adds to the confusion is that the information is subject to temporal changes. This makes it very challenging for surgeons to remain informed constantly, correctly, and completely. After all, surgeons are obliged to make the best decision for their patient, as they are the proxy for patients. How can we fulfil this role if it is so challenging to get to the correct and necessary information?

Most surgeons are aware that some valves are labelled incorrectly, but many do not realize the magnitude of this issue. The extent is hard to fathom since relevant information is confusing and often hard to find. Manufacturers should provide this kind of information, although it is of utmost importance. This article contributes to the awareness of surgeons and other practitioners. It shows the relevance and the magnitude of the problem of incorrect labelling. We measured, compared, and demonstrated the extent of this problem for the first time.

## CONCLUSION

Up until now, valve metrics in supra-annular aortic valve prostheses remained an unclear field. The paucity of relevant information on the metrics of these prostheses adds to the risk of PPM. This article gives a clear overview of all relevant metrics and warns for the confusing labelling of most of these valves. It provides very useful information for surgeons to choose and implant the correct sized aortic valve prostheses in their patients. We urge manufacturers to include this information in their publicly available information.

## Data Availability

The data underlying this article are available in the article.

## References

[1] van Boxtel A , Mariani M, Ebels T. All surgical supra-annular aortic valvar tissue prostheses are labelled too large. Interdiscip Cardiovasc Thorac Surg. 2023;36:ivad076. 37184926 10.1093/icvts/ivad076 PMC10301699

[2] ISO 5840-2:2021 (E). International Standard. *Cardiovascular Implants—Cardiac Valve Prostheses. Part 2: Surgically Implanted Heart Valve Substitutes*. Geneva: ISO, 2021.

[3] van Beek J. *Schut’s ATLAS der Geometrische Meettechniek*. Groningen: SGM, 1994:69.

[4] Gilon D , Cape EG, Handschumacher MD, et al Effect of three-dimensional valve shape on the hemodynamics of aortic stenosis: three-dimensional echocardiographic stereolithography and patient studies. J Am Coll Cardiol. 2002;40:1479-1486. 12392840 10.1016/s0735-1097(02)02269-6

[5] Garcia D , Pibarot P, Landry C, et al Estimation of aortic valve effective orifice area by Doppler echocardiography: effects of valve inflow shape and flow rate. J Am Soc Echocardiogr. 2004;17:756-765. 15220901 10.1016/j.echo.2004.03.030

[6] Saikrishnan N , Kumar G, Sawaya F, Lerakis S, Yoganathan A. Accurate assessment of aortic stenosis: a review of diagnostic modalities and hemodynamics. Circulation. 2014;129:244-253. 24421359 10.1161/CIRCULATIONAHA.113.002310

[7] Bach D. Echo/Doppler evaluation of hemodynamics after aortic valve replacement: principles of interrogation and evaluation of high gradients. JACC Cardiovasc Imaging. 2010;3:296-304. 20223428 10.1016/j.jcmg.2009.11.009

[8] Colli A , Marchetto G, Salizzoni S, et al The TRIBECA study: (TRI)fecta (B)ioprosthesis (E)valuation versus (C)arpentier Magna-Ease in (A)ortic position. Eur J Cardiothorac Surg. 2016;49:478-485. 25769464 10.1093/ejcts/ezv070

[9] Phan K , Ha H, Phan S, Misfeld M, Di Eusanio M, Yan T. Early hemodynamic performance of the third generation St Jude Trifecta aortic prosthesis: a systematic review and meta-analysis. J Thorac Cardiovasc Surg. 2015;149:1567-1575.e1-2. 25802135 10.1016/j.jtcvs.2015.01.043

[10] Duggan SM , Sadeque S, Karamanou D, et al Early Hemodynamic Performance of the crown PRT aortic prosthesis: a prospective study. J Heart Valve Dis. 2018;27:87-96. 30560604

[11] Fukuhara S , Shiomi S, Yang B, et al Early structural valve degeneration of trifecta bioprosthesis. Ann Thorac Surg. 2020;109:720-727. 31398357 10.1016/j.athoracsur.2019.06.032

[12] Head SJ , Mokhles MM, Osnabrugge RLJ, et al The impact of prosthesis-patient mismatch on long-term survival after aortic valve replacement: a systematic review and meta-analysis of 34 observational studies comprising 27 186 patients with 133 141 patient-years. Eur Heart J. 2012;33:1518-1529. 22408037 10.1093/eurheartj/ehs003

